# A review of some new or little-known species of the genus *Gnorimoschema* (Lepidoptera, Gelechiidae) from the Palaearctic region

**DOI:** 10.3897/zookeys.857.34188

**Published:** 2019-06-25

**Authors:** Oleksiy Bidzilya, Peter Huemer, Kari Nupponen, Jan Šumpich

**Affiliations:** 1 Institute for Evolutionary Ecology of the National Academy of Sciences of Ukraine, 37 Academician Lebedev str., 03143, Kiev, Ukraine Institute for Evolutionary Ecology, National Academy of Sciences of Ukraine Kiev Ukraine; 2 Tiroler Landesmuseen Betriebsges.m.b.H., Natural History Collections, Krajnc-Str. 1, A-6060 Hall in Tirol, Austria Natural History Collections Innsbruck Austria; 3 Merenneidontie 19 D, FI-02320 Espoo, Finland Unaffiliated Espoo Finland; 4 National Museum, Natural History Museum, Department of Entomology, Cirkusová 1470, CZ-193 00 Praha 9 - Horní Počernice, Czech Republic Natural History Museum Prague Czech Republic

**Keywords:** New species, new records, new synonym, systematic, distribution, brachyptery, Russia, Siberia, Tadzhikistan, Mongolia, DNA barcoding

## Abstract

Six new species of *Gnorimoschema* Busck, 1900 are described: *G.pamira***sp. nov.** (Tadzhikistan), *G.brachyptera***sp. nov.** (Russia: Buryatia), *G.altaica***sp. nov.** (Russia: Altai), *G.tabazhok***sp. nov.** (Russia, Altai, Tuva), *G.yakovlevi***sp. nov.** (Russia: Altai, Buryatia), *G.kozlovi***sp. nov.** (Mongolia). A new synonym is established: *G.mikkolai* Povolný, 1994 **syn. nov.** of *G.radkevichi* Piskunov, 1980. *Gnorimoschemamontanum* Povolný, 1966, **sp. rev., stat. nov.** is taken out from synonymy with *G.soffneri* (Riedl, 1965). An annotated check-list of the genus *Gnorimoschema* in the Palaearctic region is provided.

## Introduction

The Gnorimoschemini is an extremely species rich tribe in the subfamily Gelechiinae. Altogether about 900 species and 44 genera are known world-wide. The tribe is most diverse in the Palaearctic region, where more than 300 species from 21 genera are known (Povolný 2002; Bidzilya and Li 2010; Huemer and Karsholt 2010). The highest generic diversity is found in the Neotropics, with about 180 species known from the Nearctic region (Povolný 2002; Lee et al. 2009; Huemer and Karsholt 2010). Studies on the Oriental, Australian and Afrotropical fauna of Gnorimoschemini are rather fragmentary; however, the tribe is likely less diverse in these regions than in the Holarctic and Nearctic. Despite the progress in the study of PalaearcticGnorimoschemini in the last decades (Li and Bidzilya 2008; Huemer and Karsholt 2010; Bidzilya and Li 2016), the tribe is still in need of considerable taxonomic and faunistic study especially in central and eastern regions, where the discovery of many new taxa is expected.

The classification of the Gelechiidae is under dispute (Ponomarenko 2005; Karsholt et al. 2013). However, authors generally agree to place Gnorimoschemini in the subfamily Gelechiinae. Within this subfamily the Gnorimoschemini share with Gelechiini synapomorphy such as the conspicuous dilation of the lateral parts of the vinculum (Huemer and Karsholt 2010). Povolný (1964, 2002) did not specify autapomorphies for Gnorimoschemini, but defined the tribe mainly by a combination of genitalia characters. Currently, the monophyly of the tribe is supported by the hook-like signum and a lateral zone of microtrichia in the ostial area (Huemer and Karsholt 1999, 2010; Ponomarenko 2005).

The generic classification of Gnorimoschemini is poorly developed. A phylogeny of the tribe proposed by Povolný and Šustek (1988) and based on methods of numerical taxonomy includes seven groups of genera. These groups are rather weakly defined and their taxonomic status remains uncertain. The diagnostic characters of male and female genitalia of *Gnorimoschema* Busck, 1900 were recently discussed (Huemer and Karsholt 2010; Li and Bidzilya 2017). Within the tribe, *Gnorimoschema* is most closely related to the Nearctic genus *Neoschema* Povolný, 1967 and more distantly to the mainly Neotropical genus *Symmetrischema* Povolný, 1967 (Povolný 1991).

Most of the Palaearctic species of *Gnorimoschema* are difficult to separate from other Gnorimoschemini externally and often are confused with other species, e.g. of the genus *Scrobipalpa* Janse, 1951. However, some mainly large-sized species of *Gnorimoschema* are distinguished from other genera by the narrow elongated wings. Additionally, the males can be recognized by the characteristic shape of the uncus and terminal portion of valvae which are usually protruded and clearly visible under a binocular microscope.

The species of *Gnorimoschema* inhabit primarily open landscapes. In the Palaearctic region they are most diverse in xeromontane habitats. Several species are restricted to sand dunes and sandy riverbanks in the northern and central Palaearctic.

*Gnorimoschema* is most diverse in the Nearctic region where 95 species are known (Lee et al. 2009). The Palaearctic species of the genus were studied intensively by Povolný, who contributed tremendously to the taxonomy and diversity of the genus (Povolný 1966, 1967, 1984, 1992, 1994, 2002). After that, *Gnorimoschema* was later revised in Europe (Huemer and Karsholt 2010) and China (Li and Bidzilya 2017). As a result, twenty-one species have been recorded from the Palaearctic region, the taxonomic state of several species was clarified and some new synonymies proposed. This contribution aims to describe six additional new species from Tadzhikistan, Russia (Southern Siberia) and Mongolia, to clarify the taxonomic state of some species and to add several new country records. The description of new species is supported morphologically and, for the majority, confirmed by DNA barcodes (mtCOI gene). We also provide an annotated check-list which includes all recent changes in taxonomy and distribution of Palaearctic species of *Gnorimoschema*.

## Material and methods

### Specimens

Adults were collected by light trapping or by hand netting. Male and female genitalia were dissected and prepared using standard methods (Huemer and Karsholt 1999).

The present contribution is based on material deposited in the following collections:

**LMK** Landesmuseum Kärnten, Klagenfurt, Austria

**MZH** Finnish Museum of Natural History, Helsinki, Finland

**NMPC** National Museum Prague, Czech Republic

**NUPP** Research collection of Kari & Timo Nupponen, Espoo, Finland

**SMNK** Staatliches Museum für Naturkunde Karlsruhe, Germany

**TLMF** Tiroler Landesmuseum Ferdinandeum, Hall in Tirol, Austria

**ZIN** Zoological Institute Russian Academy of Sciences, Sankt-Petersburg, Russia

**ZMKU** Zoological Museum Kiev Taras Shevchenko National University, Ukraine

### Photographic documentation

Pinned specimens were photographed with an Olympus E-410 digital camera attached to an Olympus SZX12 microscope or with Canon 750D and MP-E-65 mm lens. Slide-mounted genitalia were photographed with a Canon EOS 600D digital camera mounted on an Olympus U-CTR30-2 trinocular head combined with a Carl Zeiss microscope body. Sets of 10–20 images were taken for each specimen and assembled to deep-focused images using Helicon Focus 6 and edited in Adobe Photoshop CS5.

### DNA Barcoding

DNA barcode sequences of the mitochondrial COI gene – a 658 base-pair long segment of the 5’ terminus of the mitochondrial COI gene (*cytochrome c oxidase 1*) – were obtained from 139 new specimens. Some specimens already sequenced, from private or published data (Mutanen et al. 2016), were also included in our dataset. DNA samples from dried legs were prepared according to prescribed standards using the high-throughput protocol of de Waard et al. (2008). Samples were processed in the Canadian Centre for DNA Barcoding (CCDB, Biodiversity Institute of Ontario, University of Guelph). Sequences were submitted to GenBank. Details of successfully sequenced voucher specimens, including complete geographic data and images, can be accessed in the Barcode of Life Data Systems (BOLD; Ratnasingham and Hebert 2007) in the public dataset “DS-LEPALGNO Lepidoptera of the Palearctic - Gelechiidae/*Gnorimoschema*” dx.doi.org/10.5883/DS-LEPALGNO.

Degrees of intra- and interspecific variation in the DNA barcode fragments were calculated under the Kimura 2 parameter (K2P) model of nucleotide substitution using analytical tools in BOLD systems v. 4.0 (http://www.boldsystems.org). A neighbour-joining tree of DNA barcode data of currently sequenced Palaearctic taxa was constructed using MEGA6 (Tamura et al. 2013) under the K2P model for nucleotide substitutions.

Furthermore, we checked the congruence of taxonomy with Barcode Index Numbers (BIN) proposed by Ratnasingham and Hebert (2013). This system clusters sequences into so-called Operational Taxonomic Units (OTUs), regardless of their previous taxonomic assignment. It is based on a two-stage algorithm that groups the sequences in a cluster and automatically assigns new sequences. All sequences > 500 bp and covering some other quality requirements are recorded independently of the project origin and assigned to a BIN (Ratnasingham and Hebert 2013). Ultimately, the BIN system is a tried and tested means of checking the concordance between morpho-taxonomically based species determinations and COI sequence data.

### Terminology

The descriptive terminology of the genitalia structures generally follows Huemer and Karsholt (2010).

## Results

### Molecular results

**Figure 1.**
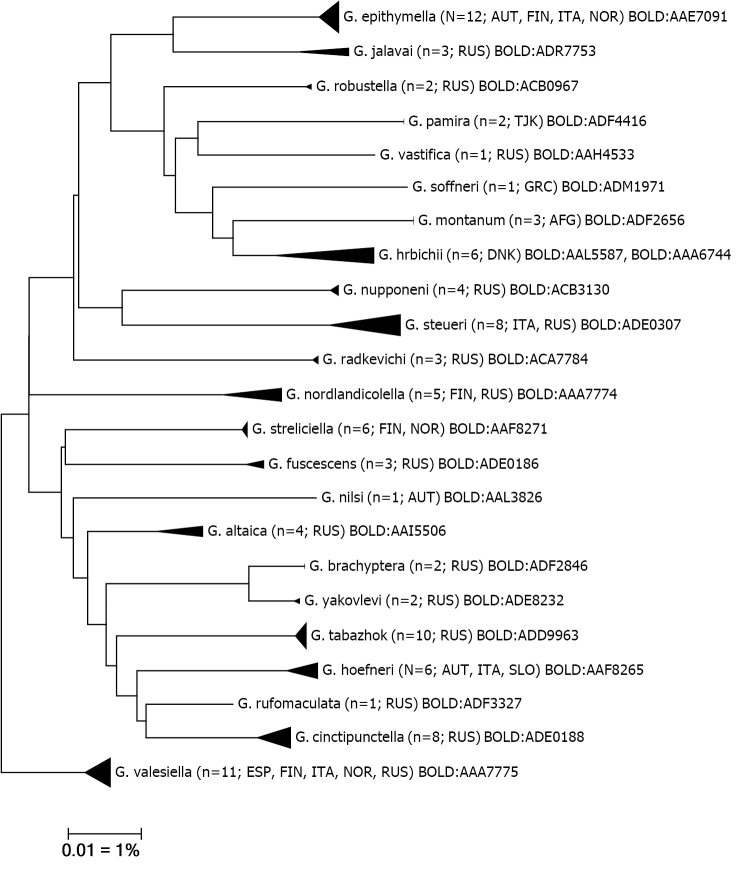
Neighbour-joining tree of Palaearctic*Gnorimoschema* species (Kimura 2 parameter). Note: the scale bar only applies to internal branches between species. The width of the triangles represents the sample size, and the height the relative genetic variation within the cluster (2× scale bar).

From 139 specimens of *Gnorimoschema* we obtained 113 sequences with 104 barcode sequences longer than 500 bp, which were used for analyses. The sequenced 23 morpho-species all group in different clusters (Fig. 1) and also can be separated at species level. Intraspecific distances range from 0% to 2.98% (mean 0.46%). Distances to the nearest neighbour vary from min. 1.39% to 6.44% (mean 3.96%) (Table 1). All successfully sequenced species, except for a single species pair (*G.brachyptera* – *G.yakovlevi*), are separated by their BINs (Barcode Index Number) in BOLD (Ratnasingham and Hebert 2013).

Further information on the genetic results can be found under each species.

**Table 1:** COI sequences of *Gnorimoschema* species in the *Palaearctic*. Intraspecific mean K2P (Kimura 2 parameter) divergences, maximum pairwise distances, and distance to the nearest neighbour in percentage.

**Species**	**Mean Intra-Sp**	**Max Intra-Sp**	**Nearest Species**	**Distance to NN**
* G. altaica *	0.63	1.32	* G. valesiella *	3.29
* G. brachyptera *	0	0	* G. yakovlevi *	1.39
* G. cinctipunctella *	0.51	0.92	* G. rufomaculata *	2.93
* G. epithymella *	0.3	0.8	* G. jalavai *	3.96
* G. fuscescens *	0.32	0.49	* G. rufomaculata *	3.6
* G. herbichii *	1.57	2.98	* G. robustella *	3.78
* G. hoefneri *	0.59	1.17	* G. rufomaculata *	3.44
* G. jalavai *	0.93	1.39	* G. epithymella *	3.96
* G. montanum *	0	0	* G. herbichii *	4.27
* G. nilsi *	N/A	0	* G. altaica *	4.93
* G. nordlandicolella *	0.69	1.6	* G. valesiella *	4.14
* G. nupponeni *	0.15	0.31	* G. valesiella *	5.6
* G. pamira *	0	0	* G. vastifica *	5.26
* G. radkevichi *	0.1	0.15	* G. valesiella *	5.57
* G. robustella *	0.15	0.15	* G. herbichii *	3.78
* G. rufomaculata *	N/A	0	* G. cinctipunctella *	2.93
* G. soffneri *	N/A	0	* G. herbichii *	4.52
* G. yakovlevi *	0.15	0.15	* G. brachyptera *	1.39
* G. steueri *	0.89	2.18	* G. nupponeni *	6.44
* G. streliciella *	0.05	0.16	* G. cinctipunctella *	4.11
* G. tabazhok *	0.16	0.39	* G. altaica *	4.11
* G. valesiella *	0.47	0.98	* G. altaica *	3.29
* G. vastifica *	N/A	0	* G. robustella *	4.91

### Descriptions of new species

#### Gnorimoschema pamira sp. nov.

Taxon classificationAnimaliaLepidopteraGelechiidae

**Figures 2–19.**
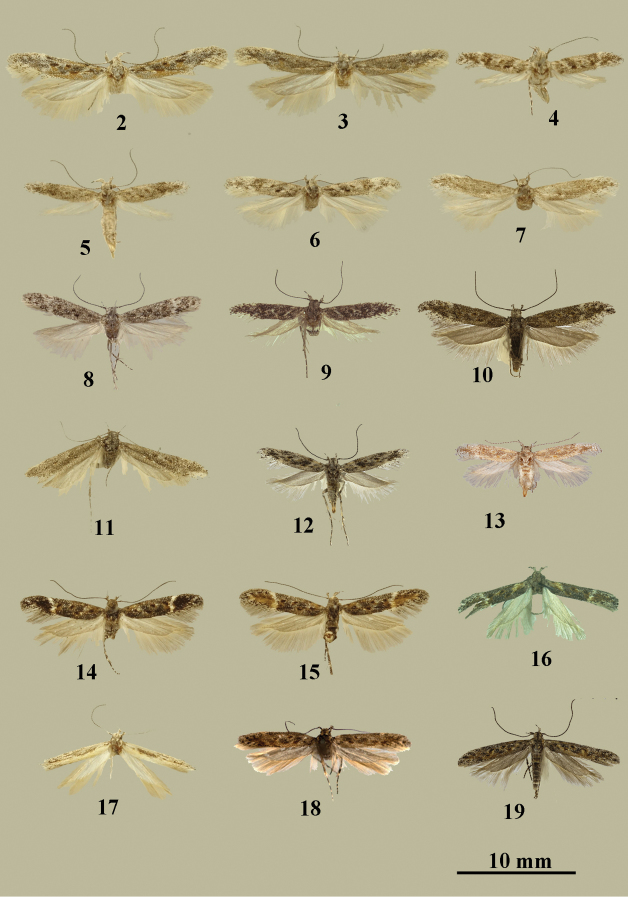
*Gnorimoschema* adults **2***G.pamira* sp. nov. – HT, male, Pamir (gen. slide 402/16, O. Bidzilya) **3***G.pamira* sp. nov. – PT, female, Pamir (gen. slide 401/16, O. Bidzilya) **4***G.brachyptera* sp. nov. – HT, female, Buryatia (gen. slide 160/16, O. Bidzilya) **5***G.brachyptera* sp. nov. – PT, female, Buryatia **6***G.brachyptera* sp. nov. – PT, male, Buryatia (gen. slide 159/16, O. Bidzilya) **7***G.brachyptera* sp. nov. – PT, male, Buryatia (gen. slide 240/16, O. Bidzilya) **8***G.altaica* sp. nov. – HT, male, Altai (gen. slide 31/18, O. Bidzilya) **9***G.altaica* sp. nov. – PT, female, Altai (gen. slide 30/18, O. Bidzilya) **10***G.tabazhok* sp. nov. – HT, male, Altai **11***G.tabazhok* sp. nov. – PT, male, Altai (gen. slide 1250, P. Huemer) **12***G.tabazhok* sp. n. – PT, female, Altai (gen. slide 18595, J. Šumpich) **13***G.tabazhok* sp. nov. – PT, female, Altai (gen. slide gen. slide GP 3_2.1.2019KN) **14***G.yakovlevi* sp. nov. – HT, male, Altai (gen. slide 406/16, O. Bidzilya) **15***G.yakovlevi* sp. nov. – PT, male, Altai **16***G.yakovlevi* sp. nov. – PT, female, Buryatia (gen. slide 69/18, O. Bidzilya) **17***G.kozlovi* sp. nov. – HT, male, Mongolia (gen. slide 236/15, O. Bidzilya) **18***G.radkevichi* Pisk. – HT, female, Mongolia **19***G.radkevichi* Pisk., male, Altai.

**Figures 20–25.**
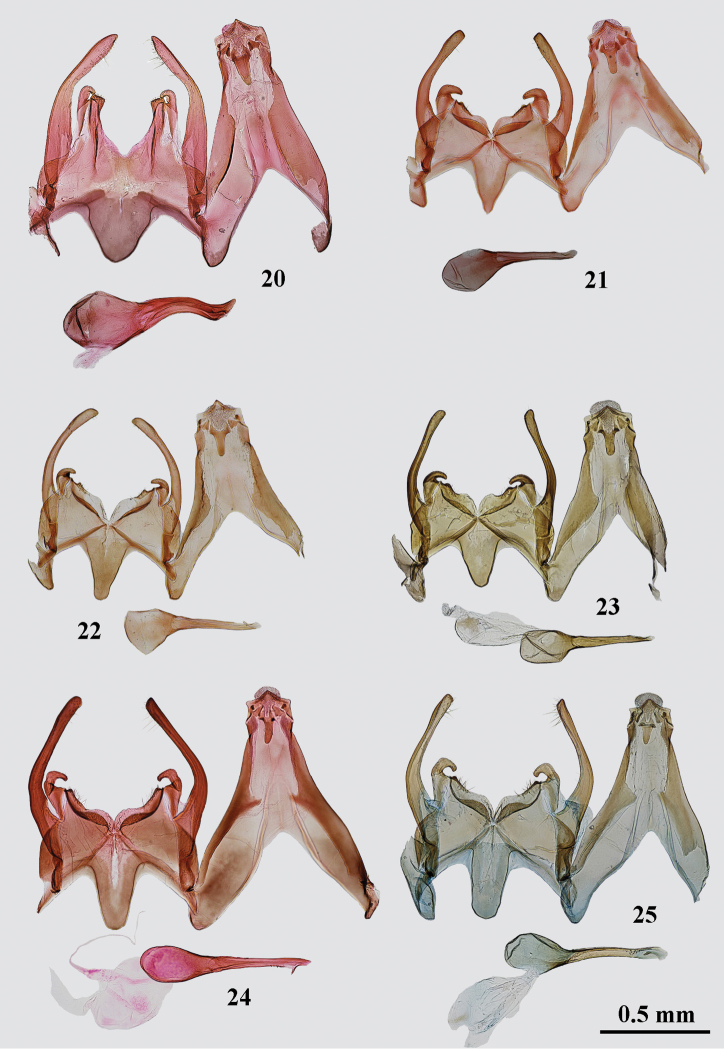
*Gnorimoschema* male genitalia **20***G.pamira* sp. nov. – HT, Pamir (gen. slide 402/16, O. Bidzilya) **21***G.brachyptera* sp. nov. – PT, Buryatia (gen. slide 159/16, O. Bidzilya) **22***G.brachyptera* sp. nov. – PT, Buryatia (gen. slide 240/16, O. Bidzilya) **23***G.altaica* sp. nov. – HT, Altai (gen. slide 31/18, O. Bidzilya) **24***G.tabazhok* sp. nov. – PT, Altai (gen. slide 1250, P. Huemer) **25***G.tabazhok* sp. nov. – PT, S Ural (gen. slide 43/18, O. Bidzilya) (gen. slide 43/18, O. Bidzilya).

Figs 2 , 3 , 20 , 34

##### Material examined.

**Holotype.** TADZHIKISTAN♂; W-Pamir Mts., Pianj/Pamir River by Zugvand village; 37°00'55"N, 72°34'32"E; 2810 m; 25 Jul. 2013; K. Nupponen & R. Haverinen leg.; gen. slide 402/16, O. Bidzilya; TLMF Lep 21646; NUPP.

**Paratypes.** 2 ♀; same data as for holotype; gen. slide 401/16, O. Bidzilya; TLMF Lep 21647; NUPP.

##### Description.

Adult (Figs 2, 3). Wingspan 15.8–16.0 mm. Head covered with white, brown-tipped scales; segment II of labial palpus white mixed with brown, inner surface white, with brush of modified scales on underside, segment III brown with white base and apex, acute, scape brown, densely mixed with white, flagellum grey, black-ringed; thorax white mottled with brown, tegulae with several brown scales; forewing covered with white, black-tipped scales, oblique narrow white fascia from about 1/8 of costal margin to 1/3 of fold, sub-costal vein mottled with brown to 2/3 length, dorsal margin brown under distal half of fold, brown spot in fold, short black streak edged with brown in mid wing, longer black streak with brown scales beneath on 2/3 length in cell, diffuse white sub-apical fascia at ¾ length, costal margin mottled white before apex, fringe white, black-tipped; hindwing and fringe light grey.

##### Variation.

The black pattern of one female paratype is more extensive making the specimen look darker, white basal fascia indistinct (Fig. 3).

*Male genitalia* (Fig. 20). Uncus sub-rectangular, apex triangular, pointed; gnathos weakly curved, gradually narrowed apically; tegumen narrow, anteromedial emargination triangular, extending to about half length of tegumen; valva broad at basal half, curved and constricted in middle, apex narrow, rounded; sacculus straight, as broad as valva in its narrowest mid length, distal portion narrow, strongly curved inwards, gap to vincular process very narrow; vinculum broad, posterior margin with deep and broad sub-ovate medial emargination, lateral process short, sub-rectangular, posterolateral corner join with the tip of sacculus; saccus broad on base, sub-triangular, apex rounded, not extended beyond top of pedunculus; phallus broad, s-curved, with pointed apex, caecum inflated, slightly exceeding half length of phallus.

*Female genitalia* (Fig. 34). Papilla analis elongate, sub-ovate, densely covered with short setae; apophysis posterioris twice longer than segment VIII; segment VIII evenly sclerotized, slightly broader than long in middle; sub-genital plates separated by narrow membranous area covered with fine microtrichia, widened anteriorly to rhomboidal, strongly edged sub-ostial membrane; anterior margin of sternum VIII with distinct triangular anteromedial projection; apophysis anterioris 2/3 length of segment VIII, curved before apex; colliculum large, sub-quadrangular, two times as broad as ductus bursae; ductus bursae narrow, about of even width; corpus bursae pear-shaped, about as long as ductus bursae, signum on the left side near entrance of corpus bursae, base small, distal hook long, narrow, nearly straight except for curved and pointed apical fifth.

##### Diagnosis.

The new species can be recognized externally by the rather contrasting, greyish-black forewings with well-developed light brown pattern along veins and near dorsal margin. *Gnorimoschemacinctipunctella* (Erschoff, 1877) is more grey, the light brown pattern is usually less extensive, but some specimens look very similar. *Gnorimoschematabazhok* is smaller in size (11.0–15.5 mm), more uniformly grey, black spots are less distinct. *Gnorimoschemaradkevichi* Piskunov, 1980 is smaller in size (12.0–14.0 mm) and has distinct black or light brown spot in the fold. The male genitalia are well recognizable by the sub-rectangular vincular process. *Gnorimoschemabodillum* Karsholt & Nielsen, 1974 is most similar to the new species regarding the male genitalia, but the vincular process is pointed, triangular rather than sub-rectangular, the sacculus is narrower, the vinculum is deeper emarginated medially and the phallus is narrower. The female genitalia are characterized by the strongly concave and well sclerotized anterior margin of sternum VIII in combination with unmodified sub-genital plate and narrow, straight with slightly curved apex of the signum. *Gnorimoschemabodillum* is similar but anterior margin of sternum VIII is less concave.

##### Molecular data.

BINBOLD:ADF4416 (n=2). The intraspecific divergence of the barcode region is 0%. The distance to the nearest neighbour *G.vastificum* Braun, 1926 is 4.2% (p-dist). This distance is the proportion (*p*) of nucleotide sites at which two sequences being compared are different. It is obtained by dividing the number of nucleotide differences by the total number of nucleotides compared. It does not make any correction for multiple substitutions at the same site, substitution rate biases, or differences in evolutionary rates among sites.

##### Distribution.

Tadzhikistan (W Pamir).

##### Biology.

Host plant unknown. Adults were collected by light in late July at an elevation of 2800 m. The collecting site is the edge between a steep rocky slope and riverside sand dunes with plenty of *Salix* (Fig. 43).

##### Etymology.

The species name, a noun in apposition, reflects the distribution of the new species in the Pamir region of Tadzhikistan.

#### Gnorimoschema brachyptera sp. nov.

Taxon classificationAnimaliaLepidopteraGelechiidae

Figs 4–7 , 21–22 , 35–36

##### Material examined.

**Holotype.** RUSSIA ♀; S-Buryatia, Hamar Daban Mts., Murtoy River, Gusinoe ozero village 6 km NW; 51°11-13'N, 106°10-12'E; 700 m; forest steppe; 27 May 2006; K. Nupponen leg.; gen. slide 160/16, O. Bidzilya; TLMF Lep 21632; NUPP.

**Paratypes.** 1 ♀, same data as for holotype; gen. slide 122/18, O. Bidzilya; TLMF Lep 21634; NUPP; 1 ♀, same data as for holotype; TLMF Lep 21633; NUPP; 1 ♂; same data as for holotype; gen. slide 159/16, O. Bidzilya; TLMF Lep 21636; NUPP; 1 ♂; same data as for holotype; gen. slide 240/16, O. Bidzilya; TLMF Lep 21635; NUPP; 1 ♂; Chita reg., 23 km N Kyra; 9 Aug. 1994; E. Ivanov leg.; gen. slide 90/15, O. Bidzilya; ZMKU; 1 ♂; same collecting data as for preceding; 10 Aug. 1994; P. Ustjuzhanin leg.; gen. slide 143/14, O. Bidzilya; ZMKU.

##### Other material.

RUSSIA 1 ♂; S-Buryatia, Hamar Daban Mnts., Murtoy River, Gusinoe ozero village 6 km NW; 51°11-13'N, 106°10-12'E, 700 m; forest steppe; 21 Jun. 2002; K. Nupponen leg.; gen. slide 194/16, O. Bidzilya; TLMF Lep 21645; NUPP.

##### Description.

Adult. *Male* (Figs 6, 7). Wingspan 12.8–13.5 mm. Head light grey, frons white; segment 2 of labial palpus white mixed with brown in distal half, inner surface white, with brush of modified scales on lower surface, segment III brown with white medial and apical rings, acute; scape brown with white apex, flagellum blackish-brown grey-ringed; thorax and tegulae covered with white brown-tipped scales; forewing brown, white oblique fascia from about 1/8 of costal margin to half length of the fold, diffuse white pattern in middle of cell, white broad subapical fascia on 3/4–4/5 length, paired black spots edged with brown in fold, small black prolonged spot mixed with brown in middle of cell, few black scales surrounded with brown in the corner of cell, fringe white, black-tipped; hindwing and fringe white.

Variation. The paratype (gen. slide 240/16, O. Bidzilya) appears uniformly brown, white markings and black spots are indistinct (Fig. 7).

*Female* (Figs 4, 5). Wingspan 11.1–11.3 mm. As male, but hindwing shortened to 2/3–3/4 length of the forewing and stronger narrowed in apical 1/3, apical excavation less distinct, abdomen longer compared to male.

*Male genitalia* (Figs 21, 22). Uncus sub-rectangular, apex triangular, pointed; gnathos weakly curved, of even width, apex rounded; tegumen moderately broad, anteromedial emargination triangular, extending to about half length of tegumen; valva broad at basal 1/3, then gradually curved, apex weakly widened, rounded; sacculus short, strongly broadened on base, distal portion narrow, curved inwards at right angle, gap to vincular process narrow, triangular; vinculum broad, posterior margin with broad, shallow sub-triangular emargination, lateral process short, hump-shaped; saccus sub-triangular, gradually narrowed towards rounded or weakly pointed apex, usually not extended beyond top of pedunculus; phallus narrow, straight, with needle-shaped, down-curved apical hook, group of short teeth before apex, caecum inflated, about 1/3 length of phallus.

Variation. Valva varies in width; saccus extended beyond tip of pedunculus in some specimens.

*Female genitalia* (Figs 35, 36). Papilla analis elongate, sub-triangular, densely covered with short setae; apophysis posterioris 2.5–3 times longer than segment VIII; segment VIII sub-quadrangular; subgenital plates medially strongly edged, separated with broadened posteriorly, membranous area covered with fine microtrichia, posterolateral sclerites sub-triangular, narrowly projecting anteromedially to the base of the apophysis anterioris, placed in middle of sternum VIII; anterior margin of sternum VIII deeply concave, strongly sclerotized, medial opening distinct; apophysis anterioris about as long or slightly longer than segment VIII, straight; colliculum as long as broad; ductus bursae narrow, of even width, but inflated before colliculum; corpus bursae egg-shaped, about as long as ductus bursae, signum near entrance of corpus bursae, base elongated, distal hook weakly curved, apically narrowed.

##### Diagnosis.

The new species can be recognized externally by the contrasting, light grey forewing with black oblique fascia at 1/3, the distinct black markings edged with light brown in cell and in the fold and the white subapical fascia at ¾. It resembles North European specimens of *G.herbichii* (Nowicki, 1864) (see Huemer and Karsholt 2010, pl. 1, fig. 2a–d) but the black markings are larger in *G.brachyptera*. The female is well-defined by the brachypterous hindwings. The female of *G.elbursicum* Povolný, 1984 differs in the less contrasting, lighter, grey rather than brown forewing, the smaller size (8.2 mm) and the considerably narrower hindwing. The male genitalia are characterized by the sacculus, which is inflated on base with distal portion inwardly curved at right angle. *Gnorimoschemafuscescens* Li & Bidzilya, 2017 differs in the larger gap between the posterior margin of the vinculum and the distal portion of the sacculus, and the valva with stronger inflated apex. *Gnorimoschemasteueri* Povolný, 1975 differs by the longer sacculus, the shorter and broader saccus and the shorter phallus. The medially placed sub-triangular posterolateral sclerites in combination with the long apophysis anterioris (1.5 times longer than length of sternum VIII) and the short signum are characteristic for the female genitalia.

##### Molecular data.

BINBOLD:ADF2846 (n=2), shared with *G.yakovlevi*. The mean intraspecific divergence of the barcode region is 0.15%. The distance to the nearest neighbour *G.yakovlevi* is 1.44% (p-dist).

##### Distribution.

Russia (Buryatia, Zabaikalskiy krai).

##### Biology.

Host plant unknown. Adults were collected in late May and August in dry steppe slopes with sparse vegetation (Fig. 44) at an elevation of 700–900 m.

##### Etymology.

The species name, an adjective is derived from the Greek *brachýs*, meaning short and the Greek *ptéryx*, meaning wing, referring to the shortened hindwing, the most characteristic feature of this species.

##### Remarks.

An additional male from South Buryatia (gen. slide 194/16, O. Bidzilya) collected in June is larger (14.2 mm) and looks lighter and brighter, having more extensive white pattern and well-developed orange-brown irroration around black spots. We have not found sufficient differences in the male genitalia between this specimen and additional males from the type-series. However, we decided to not include this specimen among the type-series due to the lack of females.

#### Gnorimoschema altaica sp. nov.

Taxon classificationAnimaliaLepidopteraGelechiidae

Figs 8 , 9 , 23 , 37

##### Material examined.

**Holotype.** RUSSIA ♂; Altai Republic, Kosh-Agach Distr., Kurai env. (15 km SW), Dzhangyzkol Lake (or Salagana Lake); 50°10'49"N, 87°44'19"E; 1830 m; coniferous forest/steppe; 24–25 Jun. 2015; J. Šumpich leg.; gen. slide 31/18, O. Bidzilya; NMPC-Lep-0313; NMPC.

**Paratypes.** 2 ♂, 1 ♀; same data as for holotype; gen. slide 30/18♀, O. Bidzilya; NMPC-Lep-0312, NMPC-Lep-0349; NMPC.

##### Other material.

RUSSIA 1♀; S-Buryatia, Hamar Daban mnts., Murtoy River, Gusinoe ozero village 6 km NW; 51°11-13'N, 106°10-12'E; 700 m; forest steppe; 27 May 2006; K. Nupponen leg.; gen. slide 232/16, O. Bidzilya; TLMF Lep 21639; NUPP.

Description. Adult. *Male* (Fig. 8). Wingspan 11.8 mm. Head covered with white, black-tipped scales, frons white; segment II of labial palpus black mixed with white, inner and upper surface white, with brush of modified scales on underside, segment III black with broad white medial ring and white apex, acute, scape black with white apex, flagellum black, white-ringed; thorax and tegulae black mixed with white; forewing covered randomly with brown and white scales, diffuse black spot mixed with brown in fold, in middle and in the corner of cell, diffuse white subapical fascia on 3/4 length, fringe white, black-tipped; hindwing and fringe light grey.

*Female* (Fig. 9). Wingspan 11.4 mm. As male, but darker, black pattern more distinct, subapical white fascia not developed, hindwing shortened to 2/3 length of forewing and stronger narrowed in apical 1/3, apical excavation less distinct than in male.

*Male genitalia* (Fig. 23). Uncus sub-rectangular, apex triangular, pointed; gnathos weakly curved, of even width, apex rounded; tegumen narrow, anteromedial emargination triangular, extending to about half length of tegumen; valva weakly broadened on base, slightly curved on 1/3, then straight, apex weakly widened, rounded; sacculus short, strongly broadened on base, distal portion narrow, curved inwards at right angle, gap to vincular process narrow, sub-triangular; vinculum broad, posterior margin with broad, shallow sub-triangular emargination, lateral process short, hump-shaped; saccus sub-triangular, gradually narrowed towards rounded apex, not extended beyond top of pedunculus; phallus narrow, straight, with needle-shaped down-curved apical hook, caecum inflated, about 2/3 length of phallus.

*Female genitalia* (Fig. 37). Papilla analis elongate, sub-triangular, densely covered with short setae; apophysis posterioris 2.5 times longer than segment VIII; segment VIII sub-quadrangular; subgenital plates medially strongly edged, separated with broadened posteriorly, membranous area covered with fine microtrichia, posterolateral sclerites hockey-stick-shaped, narrowly projecting anteromedially to the base of the apophysis anterioris, placed in middle of sternum VIII; anterior margin of sternum VIII weakly concave, strongly sclerotized, medial opening small; apophysis anterioris as long as segment VIII, straight; colliculum broader than long; ductus bursae narrow, of even width, but inflated before colliculum; corpus bursae ovate, about as long as ductus bursae, signum on the left side near entrance of corpus bursae, base elongated, distal hook strongly curved in apical portion.

##### Diagnosis.

Externally *G.altaica* is rather small, uniformly blackish-grey species with indistinct markings and diffuse white subapical fascia on the forewing in the male. *Gnorimoschemavalesiella* (Staudinger, 1877) is darker, black rather than blackish-grey, and larger in size (16–18 mm). *Gnorimoschematabazhok* is greyish brown rather than blackish grey with distinct black spots in cell, and the male is larger in size (13.5–15.5 mm). The male genitalia are similar to those of the previous species except for the shorter and broader saccus. The sub-rhomboidal, prolonged medially placed posterolateral sclerites and narrow strongly curved signum are characteristic for the female genitalia. *Gnorimoschemaepithymella* (Staudinger, 1859) differs in the narrower posterolateral sclerites, shorter and basally narrower apophysis anterioris, and weakly curved signum.

##### Molecular data.

BINBOLD:AAI5506 (n=27), shared with an unrevised species from North America. The mean intraspecific divergence of the barcode region is 0.7%, the maximum distance 2,57% (including North American specimens for the same BIN). The distance to the nearest neighbour *G.contraria* Braun, 1921 from North America is 2.57% (p-dist).

##### Distribution.

Russia (Altai).

##### Biology.

Host plant unknown. Adults were collected in late June in grassy steppe with rock protrusions at an elevation of 1800 m (Fig. 46).

##### Etymology.

The species name, a noun in apposition, reflects the distribution of the new species in the Altai Mountains of Russia.

##### Remarks.

A single female from Buryatia is very close to *G.altaica* in barcode but differs considerably in the female genitalia. Hence, we did not include this specimen among the type series. It is interesting that this female is very similar in barcode to an undescribed species of *Gnorimoschema* from USA and Canada but differs from the latter both externally and in the female genitalia (Nazari, pers. comm.).

#### Gnorimoschema tabazhok sp. nov.

Taxon classificationAnimaliaLepidopteraGelechiidae

Figs 10–13 , 24–25 , 26–29 , 38–40

##### Material examined.

**Holotype.** RUSSIA ♂; Altai Republic, Kosh-Agach District, Tašanta env. (8 km N), bellow „11. station“; 49°44'11"N, 89°20'02"E; 2280 m; rocky steppe, meadows; 1 Jul. 2015; J. Šumpich leg.; NMPC-Lep-0346; TLMF.

**Paratypes.** RUSSIA – **Altai Republic** 1 ♂; 45km N of Ulagan village, Chulyshman Valley; 51°01'03"N, 88°00'39"E; 600 m; grassy steppe, rocks; 27–28 Jun. 2015; J. Šumpich leg.; NMPC-Lep-0344; NMPK; 2 ♂, 1 ♀; Kosh-Agach District, Chagan-Uzun env., Krasnaya Gorka Hill; 50°05'00"N, 88°25'15"E; 1870 m; rocky steppe; 29 Jun. 2015; J. Šumpich leg.; NMPC-Lep-0348, NMPC-Lep-0339; gen. slide 19020, J. Šumpich; NMPC; 3 ♂; Russia, Kosh-Agach Distr., Tašanta env. (10 km SW), Ulandryk Valley; 49°40'33"N, 89°04'09"E; 2200 m; grassy steppe, rocks; 30 Jun. 2015; J. Šumpich leg.; NMPC-Lep-0345; NMPC; 4 ♂, 2 ♀; Altai Mts., Kuraisky hrebet; 50°16-20'N, 87°50-55'E; 2000-2500 m; 26 Jun. 2000; T. & K. Nupponen leg.; gen. slides 222/16 (♀), 409/16, 44/18, O. Bidzilya; 1/2.i.2019 (♂), 3/2.i.2019 (♀) K. Nupponen; NUPP; 1 ♂, same collecting data as for preceding; 27 Jun. 2000; gen. slide 1/1.i.2019 K. Nupponen; NUPP; 2 same collecting data as for preceding; 28 Jun. 2000; gen. slides 408/16, O. Bidzilya, 2/1.i.2019 K. Nupponen; NUPP; 1 ♂; same collecting data as for preceding; 30 Jun. 2000; gen. slide 196/18, O. Bidzilya; NUPP; 1 ♂; Russia, Altai Republic, Kosh-Agach distr., 10 km NE Kosh-Agach village, Kurai Mts. Range, valley of Tabazhok River; 50°05'N, 88°44'E; 2100 m; 02–04 Aug. 2016; P. Huemer & B. Wiesmair leg.; LMF 2016-020; gen. slide Gel. 1250, P. Huemer; DNA Barcode TLMF 20407; TLMF; 1 ♂; Russia, Altai Republic, Northern part of Ukok plateau, Zhumaly riber basin; 2400 m; 04–06 Aug. 2016; P. Huemer & B. Wiesmair leg.; DNA Barcode TLMF Lep 21220; TLMF; 15 ♂; Russia, Altai Republic, Kosh-Agach District, Kurai env. (15 km SW), Dzhangyskol (= Salagana) Lake; 50°10'49"N, 87°44'19"E; 1830 m; grassy steppe; 24–25 Jun. 2015; J. Šumpich leg.; NMPC-Lep-0342; gen. slide 19021, J. Šumpich; (NMPC); 3 ♂; Altai Republic, Aktash env.; 50°19'12"N, 87°36'00"E; 1400 m; grassy steppe, rocks; 21 Jun. 2015; J. Šumpich; NMPC-Lep-0343; NMPC. – **Tuva Republic** 1 ♂; 75 km NE of Kosh-Agach, Ak-Chol Lake; 50°16'43"N, 89°36'44"E; 2230 m; rocky steppe, meadows; 2–3 Jul. 2015; J. Šumpich leg.; NMPC-Lep-0333; NMPC; 2 ♂; ca. 25 km W Erzin; 50°16-20'N, 94°54'E; 1250 m; steppe/stony slopes; 7–11 Jun. 1995; J. Jalava & J. Kullberg leg.; gen. slide 319/16, 4/18, O. Bidzilya; MZH. – **Chelyabinsk region** 1 ♂; S-Ural, Cheliabinsk district, near Moskovo village; 18 Jun. 1998; T. & K. Nupponen leg.; gen. slide 43/18, O. Bidzilya; NUPP.

Description. Adult. *Male* (Figs 10, 11). Wingspan 13.5–15.5 mm. Head, thorax and tegulae covered with grey black-tipped scales, segment II of labial palpus black mixed with white, outer and upper surface white with rare black scales, with brush of modified scales on underside, segment III black mixed with white, acute, scape black with sparse white-tipped scales, flagellum brown narrowly white-ringed; forewing greyish-black, veins and fold mottled with light brown, black touch in fold, black spot surrounded with light brown in middle and in the corner of cell, diffuse white subapical fascia at 3/4, costal margin mottled with white before apex, fringe white brown-tipped; hindwing and fringe light grey.

Variation. Ground colour of the forewing varies from blackish-grey grey to dark brown depending on the amount of brown scales. A single male from South Ural is characterized by the presence of large light brown spots, whereas the blackish-grey pattern is strongly reduced in this specimen.

*Female* (Figs 12, 13). Wingspan 11.0–12.0 mm. As male, but hindwing shortened to 2/3 of the length of forewing and stronger narrowed in apical 1/3, apical excavation less distinct and abdomen longer compared to male.

Variation. Forewing varies from uniformly greyish-brown with indistinct ochreous spots similar to male to more contrast, lighter appearance, with distinct dark elongated spot in the first third (Fig. 13).

*Male genitalia* (Figs 24, 29). Uncus moderately narrow, apex triangular, pointed; gnathos short, weakly curved, narrow, of equal width, apex rounded; tegumen broad on basal half, distal half narrow, anteromedial emargination deep, triangular, extending to about half length of tegumen; valva broad in basal third, then curved, distal portion nearly of equal width, apex distinctly broadened, rounded, curved outwardly, extending the top of uncus; sacculus broad at base, distal part narrow, coiled and strongly curved inwards forming about the closed ring; vinculum broad, posterior margin with broad medial emargination and with short, rounded hump-shaped lateral process; saccus moderately narrow, weakly narrowed towards truncate apex, extended to the top of pedunculus; phallus narrow, straight, pointed, with needle-shaped down-curved apical hook, caecum rounded, 3/4 length of phallus.

Variation. The apex of the valva varies from narrow to distinctly inflated; the outer margin of the sacculus is weakly broadened in some specimens; the saccus varies from sub-triangular and apically gradually narrowed to be nearly parallel-sided and sub-rectangular with truncate apex.

*Female genitalia* (Figs 38–40). Papilla analis elongate, sub-triangular, densely covered with short setae; apophysis posterioris 2.5–3.0 times longer than segment VIII; segment VIII sub-quadrangular; subgenital plates medially strongly edged, separated with broadened posteriorly membranous area covered with fine microtrichia, posterolateral sclerites sub-triangular, narrowly projecting anteromedially to the base of the apophysis anterioris, placed near posterior margin of sternum VIII; anterior margin of sternum VIII deeply concave, strongly sclerotized, medial opening distinct; apophysis anterioris about as long or slightly longer than segment VIII, straight, broadened in basal half; colliculum as long as broad; ductus bursae narrow, of even width; corpus bursae elongated, 3 times as long as broad, about as long as ductus bursae, signum on the right side near entrance of corpus bursae, stout, base elongated, distal hook broad, weakly curved, apically pointed.

##### Diagnosis.

The new species is defined externally by the grey forewing with veins mottled with light brown and distinct black spots in the cell. It differs from *G.brachyptera* by the absence of a black fascia at ¼ and the darker forewing with more distinct brown pattern. *Gnorimoschemaradkevichi* differs in the more contrasting forewing with distinct blackish-brown spots and brown pattern along dorsal margin. *Gnorimoschemasteueri* Povolný, 1975 is very similar but can be separated by the absence of white subapical spots and the blackish-brown rather than white subapical costal margin. The male genitalia are characterized by the sacculus, which is strongly curved inwards in the apical half, forming a nearly closed ring. *Gnorimoschemahoefneri* (Rebel, 1909), *G.streliciella* (Herrich-Schäffer, 1854) and *G.rufomaculata* Li & Bidzilya, 2017 are somewhat similar in the shape of sacculus which, however, is medially not broadened, and the valva is widened towards apex in these species. The sub-triangular posterolateral sclerites placed near the posterior margin of segment VIII in combination with the stout, short and broad signum are characteristic for the female genitalia of the new species. *Gnorimoschemastreliciella* is rather similar but the signum is much more slender. *Gnorimoschemabrachyptera* and *G.altaica* differ by the shape of signum (less curved in *G.brachyptera*) and shape of posterolateral sclerites (narrow in *G.altaica*).

##### Molecular data.

BINBOLD:AAD9963 (n=10). The mean intraspecific divergence of the barcode region is 0.14%, the maximum divergence is 0.39%. The distance to the nearest neighbour, an undescribed species of *Gnorimoschema* from North America, is 3.53% (p-dist).

##### Distribution.

Russia (S Ural, Altai, Tuva).

##### Biology.

Host plant unknown. The holotype was collected in early August at an elevation of 2100 m, paratypes were collected from the second half of June to early July in various kinds of rocky steppes and in dry mountain steppes with plenty of *Artemisia* at an elevation between 600–2500 m (Figs 46–48).

##### Etymology.

The species name, a noun in apposition, refers to the type locality – Tabazhok River in the vicinity of Kosh-Agach village in the Altai Mountains.

**Figures 26–31.**
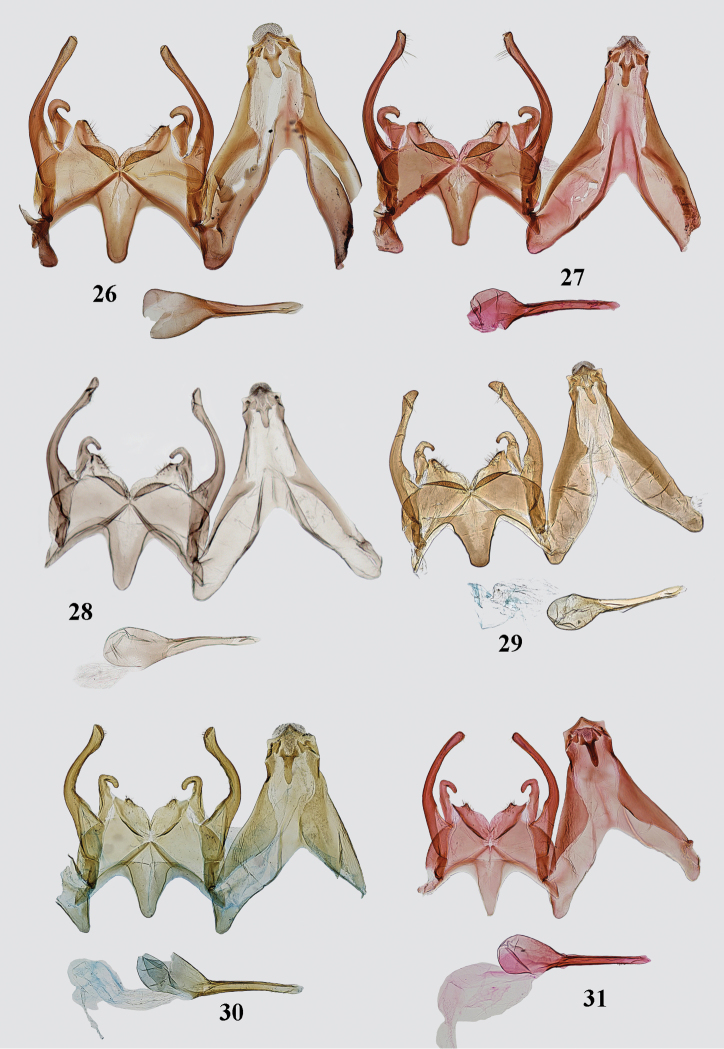
of *Gnorimoschema* male genitalia **26***G.tabazhok* sp. nov. PT, Altai (gen. slide 416/16, O. Bidzilya) **27***G.tabazhok* sp. nov. – PT, Tuva (gen. slide 319/16, O. Bidzilya) **28***G.tabazhok* sp. nov. – PT, Altai (gen. slide GP 2_1.1.2019KN) **29***G.tabazhok* sp. nov. – PT, Altai (gen. slide 19021, J. Šumpich) **30***G.yakovlevi* sp. nov. – HT, Altai (gen. slide 406/16, O. Bidzilya) **31***G.yakovlevi* sp. nov. – PT, Altai (gen. slide 1251, P. Huemer).

#### Gnorimoschema yakovlevi sp. nov.

Taxon classificationAnimaliaLepidopteraGelechiidae

Figs 14–16 , 30–31 , 41

Gnorimoschema streliciella (Herrich-Schäffer, 1854) – Li and Bidzilya 2017: 180, figs 14, 38. Misidentification.

##### Material examined.

**Holotype.** RUSSIA ♂; Altai Mts., Kuraisky hrebet; 50°16-20'N, 87°50-55'E; 2000-2500 m; 27 Jun. 2000; T. & K. Nupponen leg.; gen. slide 406/16, O. Bidzilya; TLMF Lep 21629; NUPP.

**Paratypes.** Russia – **Altai Republic** 2 ♂; Kuraisky hrebet; 50°16-20'N, 87°50-55'E; 2000–2500 m; 27 Jun. 2000; T. & K. Nupponen leg.; TLMF Lep 21630; NUPP; 5 ♂; Kosh-Agach distr., 10 km NE Kosh-Agach village, Kurai Mts. Range, valley of Tabazhok River; 50°05'N, 88°44'E; 2100 m; 02–04 Aug. 2016; P. Huemer & B. Wiesmair leg.; TLMF 2016-020; Gel. 1251♂, P. Huemer, gen. slide 432/16, O. Bidzilya; all TLMF; 1 ♂; Kosh-Agach Distr., Kurai env. (6,5 km SW); 50°10'35"N, 87°53'55"E; 1550 m; grassy steppe; 9–10 Jul. 2014; J. Šumpich leg.; NMPC. – **Buryatia Republic** 1 ♂; Hamar Daban Mts., Murtoy River, Gusinoe ozero village, 6 km NW; 51°11-13'N, 106°10-12'E; 700 m; forest steppe; 19 Jun. 2002; K. Nupponen leg.; TLMF Lep 21628; genitalia in glycerol vial; NUPP; 1 ♀; pr. Ulan-Ude, 35 km SW Ulan-Ude; 700 m; steppe hill; 17 Jul. 1996; J. Jalava & J. Kullberg leg.; gen. slide 69/18, O. Bidzilya; MZH.

##### Description.

Adult. *Male* (Figs 14–15). Wingspan 12.1–13.8 mm. Head brown, frons dirty white; segment II of labial palpus brown, outer surface white in basal 1/3–2/3, inner surface white, with brush of modified scales on underside, segment III black with white base half on lower side, acute, scape black with rare white tipped scales, flagellum blackish-brown grey-ringed; thorax and tegulae covered with brown grey-edged apically scales; forewing covered with black white-tipped scales, sub-costal vein and fold mottled with brown to half length, three black spots edged with brown in fold and in cell, black streak in base of fold, distinct white sub-apical fascia on 2/3 length, subapical 1/3 brown except for termen covered with black white-tipped scales, fringe grey; hindwing and fringe light grey.

*Female* (Fig. 16). Wingspan 11.8 mm. As male, but hindwing narrowed in apical 1/3, apical excavation less distinct compared to male.

*Male genitalia* (Figs 30–31). Uncus sub-rectangular, apex triangular, pointed; gnathos weakly curved, of even width, apex weakly pointed; tegumen moderately broad, anteromedial emargination triangular, extending to about half length of tegumen; valva broad at basal 1/3, then curved, apex rounded or weakly pointed; sacculus long, straight, as broad as valva in mid length, distal portion narrow, strongly curved inwards and down, gap to vincular process broad; vinculum broad, posterior margin with broad, shallow sub-triangular emargination, lateral process short, hump-shaped; saccus sub-triangular, apex rounded, not extended beyond top of pedunculus; phallus narrow, straight, with needle-shaped down-curved apical hook, group of short teeth before apex, caecum inflated, about 1.5 times shorter than phallus.

Variation. Distal portion of valva varies of even width or with broadened apex; saccus varies in width and length.

*Female genitalia* (Fig. 41). Papilla analis elongate, sub-triangular, densely covered with short setae; apophysis posterioris 2–2.5 times longer than segment VIII; segment VIII sub-rectangular; subgenital plates medially strongly edged, separated with broad sub-triangular membranous area covered with fine microtrichia, posterolateral sclerites large, inverted drop-shaped, narrowly projecting anteromedially, placed under mid length of posterior margin of sternum VIII; anterior margin of sternum VIII deeply concave, strongly sclerotized, medial opening small; apophysis anterioris about as long as segment VIII, strongly widened in basal 2/3, distal portion narrow, weakly curved; colliculum narrow, twice longer than broad; ductus bursae narrow, weakly broadened in anterior and posterior portion; corpus bursae sub-ovate, twice longer than broad, about as long as ductus bursae, signum near entrance of corpus bursae, base small, distal hook gradually curved, of even width except for narrowed and pointed apex, posterior margin weakly serrated.

##### Diagnosis.

The new species is recognizable by the blackish-brown forewing with distinct narrow white subapical fascia. *Gnorimoschemastreliciella* is nearly indistinguishable except for the less extensive brown pattern and the white sub-apical fascia which is usually angled towards apex. The male genitalia are characterized by the down-curved apical portion of the sacculus in combination with the moderately narrow medial emargination of the posterior margin of the vinculum. *Gnorimoschemastreliciella* differs in the broader medial emargination of the posterior margin of vinculum, and the sacculus which is broader on base, and narrower and longer in the distal portion. The large, inverted drop-shaped posterolateral sclerites in combination with the strongly concave anterior margin of sternum VIII and the apophysis anterioris distinctly widened in basal 2/3 length are characteristic for the female genitalia. *Gnorimoschemahoefneri* differs in the weakly sclerotized anterior margin of sternum VIII, the narrower apophysis anterioris and the shorter signum.

##### Molecular data.

BINBOLD:ADE8232 (n=2), shared with *G.brachyptera*. The mean intraspecific divergence of the barcode region is 0.15%. The distance to the nearest neighbour *G.brachyptera* is 1.44% (p-dist).

##### Distribution.

Russia (Altai, Buryatia).

##### Biology.

Host plant unknown. Adults were collected in semi-arid, steppe habitats with scattered vegetation (Fig. 45) from mid-June to early August up to an elevation of 2500 m.

##### Etymology.

The new species is named in honour of Prof. Roman Yakovlev (Altai State University, Barnaul, Russia) in recognition of his enormous contribution to the exploration of Lepidoptera in Altai and organization of joint expeditions.

#### Gnorimoschema kozlovi sp. nov.

Taxon classificationAnimaliaLepidopteraGelechiidae

Figs 17 , 32

##### Material examined.

**Holotype.** Mongolia ♂; Yuzhno-Gobiisky aimak, 60 km E Talyn-Bilgeh-Bulak spring; 17–19 Aug. 1969; M. Kozlov leg.; gen. slide 236/15, O. Bidzilya; ZIN.

##### Description.

Adult (Fig. 17). Wingspan 11.0 mm. Head white with several brown scales on the neck, segment II of labial palpus brown with white medial belt, upper surface white, with brush of modified scales on underside, segment III black with white medial and apical rind, acute, scape brown with few white scales on apex, flagellum brown white-ringed; thorax and tegulae covered with white brown-tipped scales; forewing yellowish cream in dorsal 1/3 width, costal 2/3 mottled with grey and brown mainly along veins, fold with indistinct light brown streak, fringe white brown-tipped; hindwing and fringe light grey.

**Figures 32–33.**
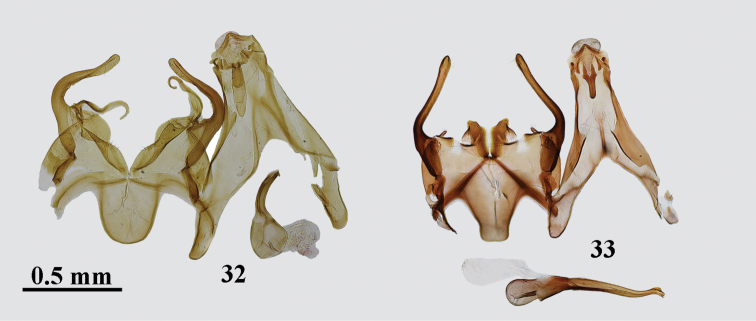
*Gnorimoschema* male genitalia **32***G.kozlovi* sp. nov. – HT, Mongolia (gen. slide 236/15, O. Bidzilya) **33***G radkevichi* Pisk. – Altai (gen. slide 441/16, O. Bidzilya).

*Male genitalia* (Fig. 32). Uncus moderately narrow, apex triangular, pointed; gnathos long, weakly curved, broadest in middle; tegumen broad in basal half, distally nearly parallel-sided, anteromedial emargination deep, triangular, extending to half length of tegumen; valva broad in basal third, strongly curved before middle, then narrow, weakly sinuate, about of equal width, apex slightly broadened and rounded, not extending the top of uncus; sacculus very long, extending nearly to the top of valva, broad on base, distal portion narrow, with pointed, coiled and downwards curved 1/4; vinculum broad, posterior margin with deep and broad sub-triangular medial emargination, with triangular lateral process and broad membranous lobe; saccus sub-rectangular, weakly narrowed towards rounded apex, not extended beyond top of pedunculus; phallus moderately broad, gradually curved, with small triangular apical hook, caecum twice shorter than the length of phallus.

*Female genitalia*. Unknown.

##### Diagnosis.

The new species is characterized by the forewing colour divided into dark brown costal and yellowish-cream dorsal parts. The male genitalia are characterized by a very long sacculus that reaches about ¾ length of valva and within the Palaearctis *Gnorimoschema*-species unique phallus with gradually curved distal portion.

##### Molecular data.

Unavailable due to lack of suitable, fresh material.

##### Distribution.

Mongolia.

##### Biology.

Host plant unknown. The holotype was collected in mid-August.

##### Etymology.

The species is named in honour of the Russian hymenopterist and well-known specialist in the family Scelionidae, Mikhail Alekseevich Kozlov, the collector of the holotype of the new species.

### Check-list of the genus *Gnorimoschema* in the Palaearctic region

New regional records are marked with an asterisk *.

### *Gnorimoschemasoffneri* (Riedl, 1965)

*Lerupsiasoffneri* Riedl, 1965: 61–62, 80.

*Gnorimoschemaantiquum* Povolný, 1967: 400, figs 5, 22–24, 41. – Karsholt and Nielsen 1974: 91; Huemer and Karsholt 2010: 38.

**Distribution.** South Europe from Spain to Bulgaria, Turkey, Iraq (Huemer and Karsholt 2010).

### *Gnorimoschemamontanum* Povolný, 1966 sp. rev., stat. n.

*Gnorimoschemaantiquummontanum* Povolný, 1966: 402, fig. 6.

*Gnorimoschemasoffnerimontanum* Povolný, 1966 – Huemer and Karsholt 2010: 38–40.

**Remarks.***Gnorimoschemaantiquummontanum* was described from the mountains of Afghanistan. It is characterized by its uniformly coloured yellowish to ochreous brown forewing with grey irroration along the veins and costal margin. The status of this taxon was recently discussed, and it was suggested that *G.montanum* may be a separate species that differs from the related *G.soffneri* and *G.antiquum* by details of the genitalia of both sexes (Li and Bidzilya 2017: 176, figs 7, 31, 32, 54). This suggestion is partially confirmed by DNA barcodes from material collected in Afghanistan which clearly separates *G.montanum* from *G.soffneri* (see Table 1). Even though the sequences are not yet known for *G.antiquum*, we consider the existing evidence sufficient to recognize *G.montanum* as a valid species.

**Distribution.** Uzbekistan, Iran, Afghanistan (Povolný 2002; Li and Bidzilya 2017).

**Figures 34–36.**
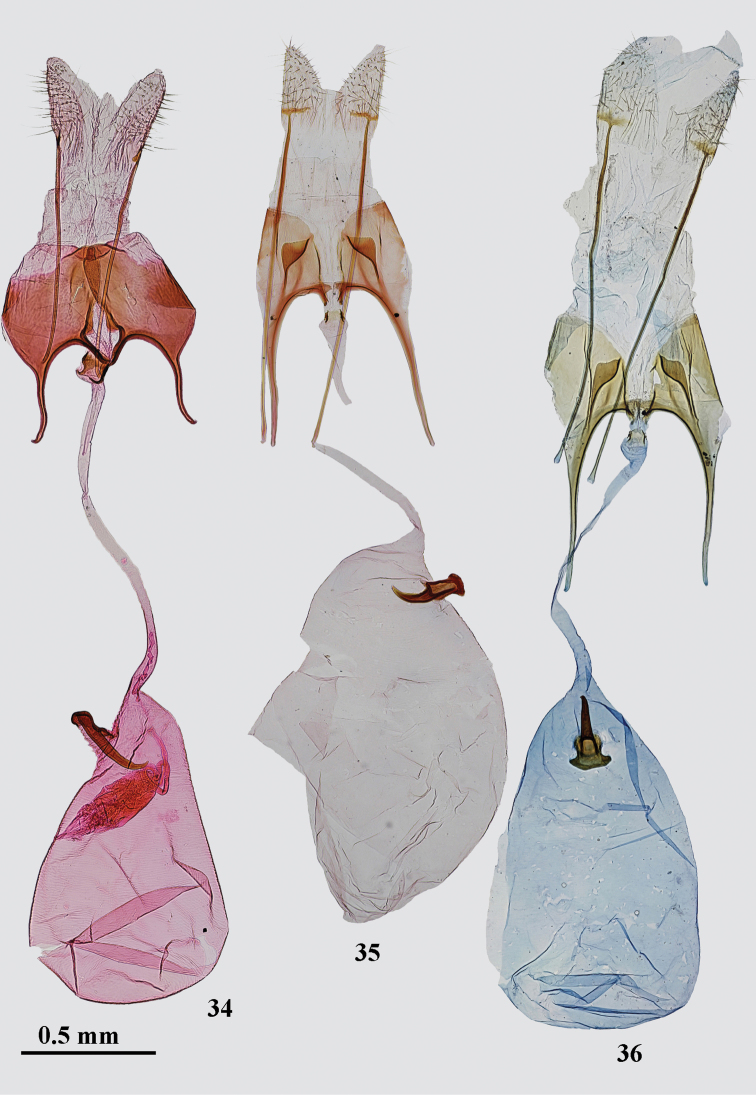
*Gnorimoschema* female genitalia **34***G.pamira* sp. nov. – PT, Pamir (gen. slide 401/16, O. Bidzilya) **35***G.brachyptera* sp. nov. – HT, Buryatia (gen. slide 160/16, O. Bidzilya) **36***G.brachyptera***sp. nov.** – PT, Buryatia (gen. slide 122/18, O. Bidzilya).

### *Gnorimoschemaherbichii* (Nowicki, 1864)

*Gelechiaherbichii* Nowicki, 1864: 17, pl. 1, fig. 6.

*Litapusillella* Rebel, 1893: 47.

Gelechia (Lita) tengstroemiella Joannis, 1910: 296. – Povolný 1964: 337.

*Litapazsiczkyi* Rebel, 1913: 173. – Povolný 1964: 337.

*Litaparentesella* Toll, 1936: 407, pl. 49, fig.18.

*Phthorimaeatengstroemi* Hackman, 1946: 61, figs. 2, 5. – Povolný 1964: 337.

*Gnorimoschemaherbichi* [sic] *mongoliae* Povolný, 1973: 19, figs. 4, 14, 22. – Li and Bidzilya 2017: 175.

*Gnorimoschemaherbichi* [sic] *kamchaticum* Povolný, 1977: 218, fig. 14. – Li and Bidzilya 2017: 175.

**Distribution.** Europe from Spain to Belarus, European part of Russia (Kirov region, Udmurtia Republic), Turkmenistan, Uzbekistan, Iraq, Mongolia, Asian part of Russia (Irkutsk region, Buryatia, Zabaikalskiy krai, Chukchi AR, Kamchatka), China (Hebei, Inner Mongolia, Ningxia, Shaanxi, Xinjiang), Canada (Alberta, Yukon, Manitoba) (Povolný 2002; Ponomarenko 2008; Falkovitsh and Bidzilya 2009; Huemer and Karsholt 2010; Nazari and Landry 2012; Bidzilya and Li 2017; Piskunov and Derzhinsky 2018).

**Figures 37–40.**
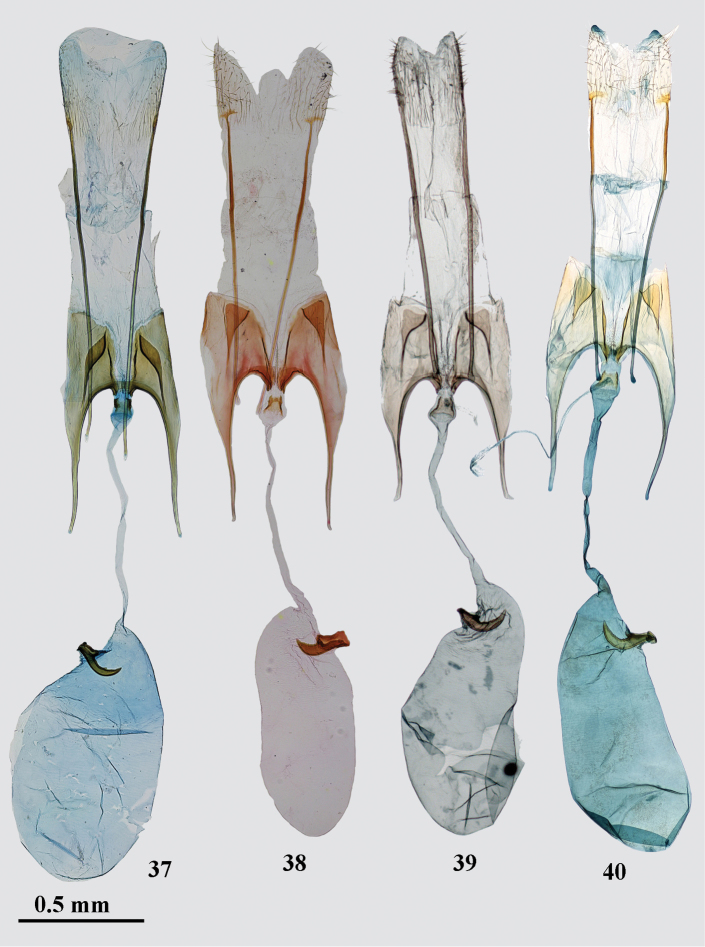
*Gnorimoschema* female genitalia **37***G.altaica* sp. nov. – PT, Altai (gen. slide 30/18, O. Bidzilya) **38***G.tabazhok* sp. nov. – PT, Altai (gen. slide 222/16, O. Bidzilya) **39***G.tabazhok* sp. nov. – PT, Altai (gen. slide gen. slide GP 3_2.1.2019KN) **40***G.tabazhok* sp. nov. – PT Altai, (gen. slide 18595, J. Šumpich).

### *Gnorimoschemabodillum* Karsholt & Nielsen, 1974

*Gnorimoschemabodillum* Karsholt & Nielsen, 1974: 91, figs 1–9.

**Distribution.** Denmark, Germany (Huemer and Karsholt 2010). A record form Taymyr Peninsula of Russia (Bidzilya 2005: 14) should most likely be referred to *G.vastificum* (Kullberg et al. 2013: 130).

### *Gnorimoschemavastificum* Braun, 1926

*Gnorimoschemavastificum* Braun, 1926: 47.

**Distribution.** Russia (Arkhangelsk region: Nenetz Autonomous Okrug, Taymyr Peninsula (?)) (Bidzilya 2005; Kullberg et al. 2013), Canada (Northwest Territories, Alaska, Yukon, Alberta, Saskatchewan, Manitoba) (Nazari and Landry 2012), USA (Utah, California) (Powell and Povolný 2001).

### *Gnorimoschemapamira* sp. nov.

**Distribution.** Tadzhikistan.

### *Gnorimoschemacinerella* Li & Bidzilya, 2017

*Gnorimoschemacinerella* Li & Bidzilya, 2017: 177, figs 8, 33.

**Distribution.** China (Yunnan) (Li and Bidzilya 2017).

### *Gnorimoschemagilvella* Li & Bidzilya, 2017

*Gnorimoschemagilvella* Li & Bidzilya, 2017: 177, figs 9, 55.

**Distribution.** China (Ningxia) (Li and Bidzilya 2017).

### *Gnorimoschemanupponeni* Huemer & Karsholt, 2010

*Gnorimoschemanupponeni* Huemer & Karsholt, 2010: 26.

**Distribution.** Ukraine (Crimea), Russia (Orenburg region) (Huemer and Karsholt 2010), Kazakhstan*.

**New records.** KAZAKHSTAN 3 ♂; North Mugozhary Mts., Altyndy village 5 km W; 48°55'29"N, 58°18’49"E; 470–520 m; 6 Sep. 2012; K. Nupponen leg.; NUPP.

### *Gnorimoschemajalavai* Povolný, 1994

*Gnorimoschemajalavai* Povolný, 1994: 57, figs 1, 6.

**Distribution.** Russia (Altai, Tuva, Irkutsk region, Buryatia, Zabaikalskiy krai, Chukchi AR (Povolný 2002; Ponomarenko 2008), Canada (Yukon) (Landry et al. 2013: 39).

**Figures 41–42.**
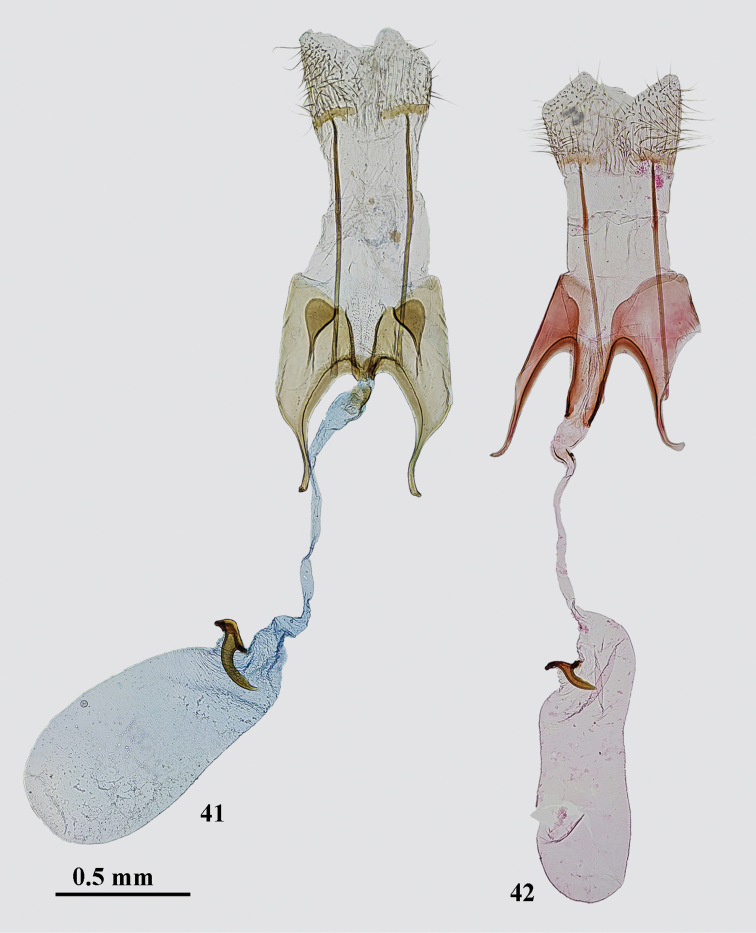
*Gnorimoschema* female genitalia **41***G.yakovlevi* sp. nov. – PT, Buryatia (gen. slide 69/18, O. Bidzilya) **42***G.radkevichi* Pisk. – Buryatia (gen. slide 303/16, O. Bidzilya).

### *Gnorimoschemarobustella* (Staudinger, 1871)

*Gelechiarobustella* Staudinger, 1871: 312.

*Phthorimaeasyrphetopa* Meyrick, 1926: 278. – Povolný 1992: 230.

**Distribution.** Russia (Arkhangelsk region, Saratov region, Volgograd region, Orenburg region, South of Krasnoyarskiy krai*) (Anikin and Piskunov 1995; Junnilainen et al. 2010; Kozlov et al. 2014); West Kazakhstan (Uralsk, Indersk Lake) (Huemer and Karsholt 2010).

**New record.** RUSSIA 1 ♂; [Krasnoyarskiy krai] Minusinsk; 5 Jul. 1924; N. Filipjev leg.; gen. slide 182/18, O. Bidzilya; ZMKU.

### *Gnorimoschemasteueri* Povolný, 1975

*Gnorimoschemasteueri* Povolný, 1975: 190, figs 1–3, 6–9.

**Distribution.** France, Italy, Germany, Austria, Czech Republic, Slovakia (Huemer and Karsholt 2010), Russia (Altai*, Krasnoyarskiy kray, Zabaikalskiy krai) (Bidzilya 2005; Akulov et al. 2018).

**New records.** RUSSIA 42 ♂; Altai Republic, Kosh-Agach distr., 17 km NNE Kokorya village, Chikhacheva Mts. Range, Talduair Mt., valley of Sajlyugem River; 50°01'N, 89°14'E; 2200 m; 30 Jul.–2 Aug. 2016; P. Huemer & B. Wiesmair; gen. slides Gel. 1247, P. Huemer; 417/16; 421/16; 423/16, 426/16, 431/16, O. Bidzilya; TLMF; 4 ♂; Altai Republic, Kosh-Agach distr., 10 km NE Kosh-Agach village, Kurai Mts. Range, valley of Tabazhok River; 50°05'N, 88°44'E; 2100 m; 2–4 Aug. 2016; P. Huemer & B. Wiesmair; TLMF 2016-020; gen. slide 411/16; 424/16; 425/16, O. Bidzilya; TLMF.

### *Gnorimoschemafuscescens* Li & Bidzilya, 2017

*Gnorimoschemafuscescens* Li & Bidzilya, 2017: 178, figs 11–13, 35–37, 57.

**Distribution.** Russia (Altai, Zabaikalskiy krai), Kyrgyzstan, Mongolia, China (Gansu, Inner Mongolia) (Li and Bidzilya 2017).

### *Gnorimoschemabrachyptera* sp. nov.

**Distribution.** Russia (Buryatia, Zabaikalskiy krai).

### *Gnorimoschemaaltaica* sp. nov.

**Distribution.** Russia (Altai).

### *Gnorimoschematabazhok* sp. nov.

**Distribution.** Russia (S Ural, Altai, Tuva).

### *Gnorimoschemaelbursicum* Povolný, 1984

*Gnorimoschemaelbursicum* Povolný, 1984: 264, fig. 1.

**Distribution.** Iran (Elburs Mts.c., Kendevan Pass).

**Remarks.** The species is known from a single brachypterous female, with genitalia characterized by the unmodified and evenly sclerotized segment VIII (Povolný 1984, 2002: pl. 1, fig. 8; pl. 60, fig. 544).

### *Gnorimoschemaepithymella* (Staudinger, 1859)

*Gelechiaepithymella* Staudinger, 1859: 242.

*Phthorimaeabrunneomaculella* Hackman, 1946: 60, figs 3, 6.

*Phthorimaeaboernii* Amsel, 1952: 123, fig. 29.

*Gnorimoschemaepithymellumkirgisicum* Povolný, 1994: 61, figs 3, 8. Subspecies.

**Distribution.** Europe from Spain to Kola Peninsula, Volga region and Western Caucasus of Russia (Kozlov and Kullberg 2006; Ponomarenko 2008; Karsholt and Huemer 2010), Algeria, Kyrgyzstan (Povolný 2002), Zabaikalskiy krai of Russia (Bidzilya 2005: 15).

### *Gnorimoschemanilsi* Huemer, 1996

*Gnorimoschemanilsi* Huemer, 1996: 78, figs 1, 3, 5, 6, 11, 12, 17, 18, 21, 22.

*Gnorimoschemanordlandicolellum* (Strand, 1902). – Povolný 1998: 337; 2002: 24.

*Gnorimoschemanilsi* Huemer, 1996. – Huemer and Karsholt 2010: 49.

**Distribution.** Austria, France, Italy (Huemer and Karsholt 2010).

### *Gnorimoschemanordlandicolella* (Strand, 1902)

Gelechia (Lita) nordlandicolella Strand, 1902: 21.

*Gnorimoschemanordlandicolella* (Strand, 1902). – Povolný 1966: 397.

*Gnorimoschemanordlandicolellaeucausta* (Meyrick, 1929). – Povolný 1967: 77.

*Phthorimaeaceceonodes* Meyrick, 1924: 278. – Povolný 1992: 230.

*Phthorimaeaeucausta* Meyrick, 1929: 492. – Povolný 1992: 230.

*Phthorimaeafennicella* Hackman, 1946: 60, figs 1, 4. – Povolný 1992: 230

**Distribution.** Northern Europe, Turkey, Uzbekistan, mountains of SE Kazakhstan, Kyrgyzstan, Afghanistan*, Russia (Altai, Irkutsk Region, Zabaikalskiy krai, Yakutia) China (Xinjiang) (Povolný 2002; Ponomarenko 2008; Huemer and Karsholt 2010; Bidzilya 2012; Li and Bidzilya 2017).

**New record.** AFGHANISTAN 1 ♂; Salang-Pass, N-Seite; 2100 m; 5–11 Jul. 1966; H. Amsel leg.; gen. slide 22/18, O. Bidzilya; SMNK.

### *Gnorimoschemastreliciella* (Herrich-Schäffer, 1854)

[no genus] *streliciella* Herrich-Schäffer, 1853: pl. 67, fig. 495.

*Gelechiastreliciella* Herrich-Schäffer, 1854: 171.

**Distribution.** Northern and parts of Central Europe (Huemer and Karsholt 2010), Russia (Middle Volga region, Buryatia), China (Inner Mongolia) (Li and Bidzilya 2017).

### *Gnoriomoschemayakovlevi* sp. nov.

**Distribution.** Russia (Altai, Buryatia).

### *Gnorimoschemahoefneri* (Rebel, 1909)

Gelechia (Lita) hoefneri Rebel, 1909: 331.

*Gnorimoschemastreliciellahoefneri* (Rebel 1909). – Povolný 2002: 25.

*Gnorimoschemahoefneri* (Rebel, 1909). – Huemer and Karsholt 2010: 53.

**Distribution.** Italia, Austria, Slovenia (Huemer and Karsholt 2010).

### *Gnorimoschemavalesiella* (Staudinger, 1877)

*Litavalesiella* Staudinger, 1877: 205.

*Gnorimoschemavalesiellacharcotti* (Meyrick, 1934). – Povolný 1967: 74.

*Litadiabolicella* Hartig, 1924: 81. – Povolný 1992: 232.

*Phthorimaeacharcoti* Meyrick, 1934: 59. – Povolný 1992: 232.

*Phthorimaeahackmani* Schantz, 1952: 19. – Povolný 1992: 232.

**Distribution.** Spain, France, Italy, Switzerland, Austria, Island, Norway, Sweden, Finland, Latvia, Caucasus, Greenland (Huemer and Karsholt 2010), Russia (Kola Peninsula, Altai, Tuva, Buryatia, Zabaikalskiy krai) (Ponomarenko 2008).

### *Gnorimoschemacinctipunctella* (Erschoff, 1877)

*Gelechiacinctipunctella* Erschoff, 1877: 344.

*Gnorimoschemacinctipunctella* (Erschoff, 1877). – Piskunov 1988: 362, figs 4, 5.

*Gnorimoschemastreliciellacinctipunctella* (Erschoff, 1877). – Povolný 1992: 232, fig. 11. pl. 3, fig. 4.

*Gnorimoschemastreliciella* (Erschoff, 1877). – Ponomarenko 2008: 328.

*Gnorimoschemamongolorum* Povolný, 1969: 4, pls 1–5, figs 1–10; pl. 32, fig. 31. – Li and Bidzilya 2017: 180.

**Distribution.** Russia: South Ural (Junnilainen et al. 2010), Altai, South of Krasnoyarskiy krai, Zabaikalskiy krai, Amur Region (Povolný 2002; Ponomarenko 2008; Bidzilya 2009), Mongolia, China (Gansu, Hebei, Inner Mongolia, Ningxia and Qinghai) (Li and Bidzilya 2017).

### *Gnorimoschemarufomaculata* Li & Bidzilya, 2017

*Gnorimoschemarufomculata* Li & Bidzilya, 2017: 183, figs 21, 22, 46–48, 62, 63.

**Distribution.** Russia (Buryatia*, Zabaikalskiy krai), China (Ningxia and Inner Mongolia Autonomous Regions), South Korea (Li and Bidzilya 2017).

**New records.** RUSSIA 3 ♂; S-Buryatia, Hamar Daban Mts., Murtoy River, Gusinoe ozero village 6 km NW; 51°11-13'N, 106°10-12'E; 700 m; forest steppe; 19 Jun. 2002; K. Nupponen leg.; gen. slide 174/16, O. Bidzilya; NUPP.

### *Gnorimoschemapiskunovi* Li & Bidzilya, 2017

*Gnorimoschemapiskunovi* Li & Bidzilya, 2017: 184, figs 23, 24, 64, 65.

**Distribution.** China (Hebei, Shanxi) (Li and Bidzilya 2017).

### *Gnorimoschemakozlovi* sp. nov.

**Distribution.** Mongolia.

### *Gnorimoschemaradkevichi* Piskunov, 1980

*Gnorimoschemaradkevichi* Piskunov, 1980: 388, figs 6, 7.

*Gnorimoschemamikkolai* Povolný, 1994: 60, figs 2, 7. Syn. nov.

**Material examined.** Holotype of *G.radkevichi*: MONGOLIA ♂; G. Alt. aim., Dutin Daba, 37 km ENE Tsogt; 14 Jul. 1970; malaise trap; V. Zaitzev & E. Narchuk leg.; Mikr. Prep. № 14777; ZIN; RUSSIA 1 ♀; Buryatia, pr. Ulan-Ude; 35 km SW Ulan-Ude; 17 Jul. 1996; 700 m; steppe hill; J. Jalava & J. Kullberg leg.; gen. slide 303/16, O. Bidzilya; MZH; 6 ♂; Altai Republic, Kosh-Agach distr., 10 km NE Kosh-Agach village, Kurai Mts. Range, valley of Tabazhok River; 50°05'N 88°44'E; 2100 m; 2–4 Aug. 2016; P. Huemer & B. Wiesmair leg.; TLMF 2016-020; gen. slide 428/16; 433/16; 441/16, O. Bidzilya; TLMF; 1 ♂; same collecting data as for preceding; genitalia in glycerol vial; TLMF; 2 ♂, Altai Republic, Aktash village, 50°19'N, 87°36'E; 1400 m; grassy steppe, rocks; 11 Jul. 2014; NMPC-Lep-0337; J. Šumpich leg.; NMPC.

**Remarks.***Gnorimoschemaradkevichi* was described from a single male (Fig. 18) collected in Mongolia: pass Dutiin-Daba in Gobi-Altai aimak, 37 km ENE of Tsogt. *Gnorimoschemamikkolai* was described from a single female collected in Magadan region of Russia: Upper Kolyma River, steppe slopes near Vetrennyi. A female from Buryatia matches the genitalia (Fig. 42) of the holotype of *G.mikkolai*. Males from Altai are identical both externally (Fig. 19) and in the genitalia (Fig. 33) to the holotype of *G.radkevichi* and fully correspond in DNA barcodes with the female holotype of *G.mikkolai* which is therefore formally synonymized with *G.radkevichi*.

**Distribution.** Russia (Altai*, Buryatia*, Magadan region), Mongolia (Piskunov 1980; Povolný 1994).

**Figures 43–48.**
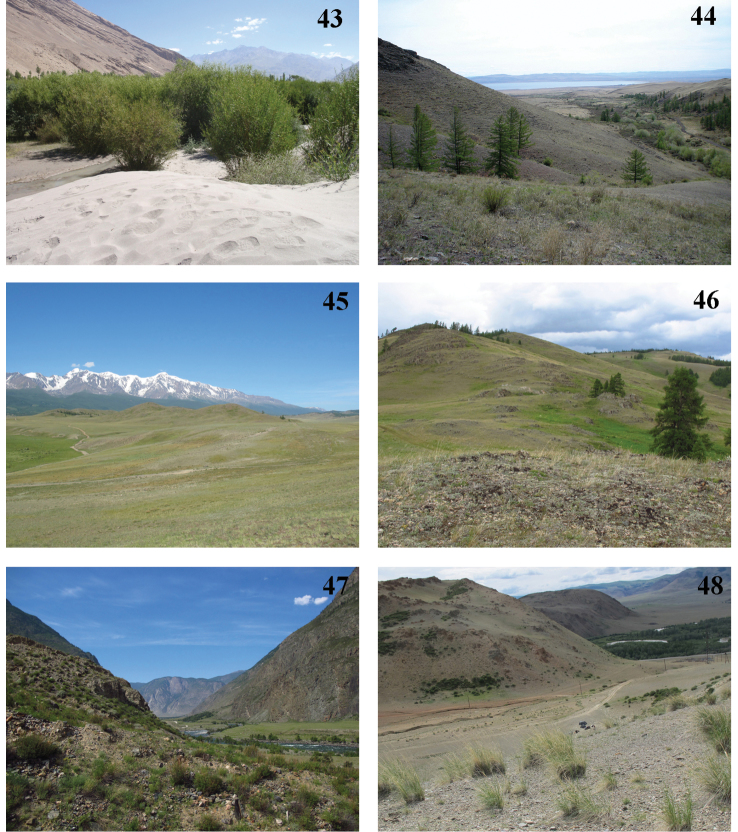
*Gnorimoschema* habitats **43** Tadzhikistan, Pamir by Zugwand, habitat of *G.pamira* sp. nov. **44** Russia, Buryatia, Gusinoe Ozero, habitat of *G.brachyptera* sp. nov. **45** Russia, Altai Mts., steppe near Kurai, habitat of *G.yakovlevi* sp. nov. **46** Russia, Altai Mts., Kurai District, steppe in the surroundings of Dzhangyskol (= Salagana) Lake, habitat of *G.altaica* sp. nov. and *G.tabazhok* sp. nov. **47** Russia, Altai Mts., Ulagan District, Chulyshman Valley, habitat of *G.tabazhok* sp. nov. **48** Russia, Altai Mts., Russia, Altai Mts., Krasnaya Gorka Hill, near Chagan-Uzun, habitat of *G.tabazhok* sp. nov.

## Supplementary Material

XML Treatment for Gnorimoschema pamira

XML Treatment for Gnorimoschema brachyptera

XML Treatment for Gnorimoschema altaica

XML Treatment for Gnorimoschema tabazhok

XML Treatment for Gnorimoschema yakovlevi

XML Treatment for Gnorimoschema kozlovi
